# Transplantation of mesenchymal stem cells for prevention of acute myocardial infarction induced heart failure: study protocol of a phase III randomized clinical trial (Prevent-TAHA8)

**DOI:** 10.1186/s13063-022-06594-1

**Published:** 2022-08-04

**Authors:** Armin Attar, Ahmad Monabati, Mohammad Montaseri, Massoud Vosough, Seyed Ali Hosseini, Javad Kojouri, Alireza Abdi-Ardekani, Peyman Izadpanah, Negar Azarpira, Gholamreza Pouladfar, Mani Ramzi

**Affiliations:** 1 Department of Cardiovascular Medicine, TAHA Clinical Trial Group, Shiraz University of Medical Sciences, Zand Street, Shiraz, 71344-1864 Iran; 2 Hematology Research Center, Shiraz University of Medical Sciences, Shiraz, Iran; 3 Department of Pathology, Shiraz University of Medical Sciences, Shiraz, Iran; 4 Student Research Committee, Shiraz University of Medical Sciences, Shiraz, Iran; 5 Department of Regenerative Medicine, Cell Science Research Center, Royan Institute for Stem Cell Biology and Technology, ACECR, Tehran, Iran; 6 Transplant Research Center, Shiraz University of Medical Sciences, Shiraz, Iran; 7 Clinical Microbiology Research Center, Nemazee Hospital, Shiraz University of Medical Sciences, Shiraz, Iran

**Keywords:** Regenerative medicine, Cell therapy, Myocardial infarction, Mesenchymal stem cells, Intracoronary injection, Acute myocardial infarction

## Abstract

**Background:**

Results from recent clinical trials on bone marrow mononuclear cell (BM-MNC) transplantation show that this intervention can help reduce the incidence of heart failure (HF) after acute myocardial infarction (AMI). However, no study has evaluated the effect of the transplantation of mesenchymal stem cells (MSCs) on a clinical endpoint such as HF.

**Methods:**

This single-blinded, randomized, multicenter trial aims to establish whether the intracoronary infusion of umbilical cord-derived Wharton’s jelly MSCs (WJ-MSCs) helps prevent HF development after AMI. The study will enroll 390 patients 3 to 7 days following AMI. Only patients aged below 65 years with impaired LV function (LVEF < 40%) will be included. They will be randomized (2:1 ratio) to either receive standard care or a single intracoronary infusion of 10^7^ WJ-MSCs. The primary outcome of this study is the assessment of HF development during long-term follow-up (3 years).

**Discussion:**

Data will be collected until Nov 2024. Thereafter, the analysis will be conducted. Results are expected to be ready by Dec 2024. We will prepare and submit the related manuscript following the CONSORT guidelines. This study will help determine whether or not the infusion of intracoronary WJ-MSCs in patients with AMI will reduce the incidence of AMI-induced HF.

**Trial registration:**

ClinicalTrials.gov NCT05043610, Registered on 14 September 2021 - retrospectively registered.

**Supplementary Information:**

The online version contains supplementary material available at 10.1186/s13063-022-06594-1.

## Introduction

Myocardial infarction (MI) represents a leading cause of mortality worldwide [1]. With a reduction in the rate of mortality due to MIs in recent decades, the incidence of heart failure (HF) has been on the rise [2]. This incidence ranges between 14 and 36% among those hospitalized due to an acute MI (AMI) [3]. HF exerts a considerable effect on healthcare systems in America, accounting for 6 million cases, 300,000 deaths, and roughly 40 billion USD worth of costs every year [4].

Despite the therapeutic efforts [5], post-MI HF still leads to a high rate of morbidities and mortalities [6, 7]. Although we have been successful in prolonging the life of HF patients and relieving symptoms, we are yet to regenerate the infarcted cardiac tissues. Hence, a gap exists in the literature as restoring the standard histological architecture of the heart should theoretically lead to improved outcomes for patients with MI-induced HF [6]. This may be possible using stem cell-based therapies [8].

### Cell-based therapy in cardiovascular disease

Toward the close of the 20th century, scientists signaled a new era in cardiovascular disease treatment through preclinical investigations in which skeletal myoblasts [9] and fetal cardiomyocytes [10] were transplanted into ischemic myocardium. Afterward, the intracardiac implantation of bone marrow (BM) cells was assessed in murine MI models [11, 12]. Human studies commenced following the turn of the century, with skeletal myoblasts being used in HF patients in 2001 [13] and BM cells being used for AMI patients in 2002 [14]. From then on, numerous investigations have aimed to amend the cardiovascular damage caused by diseases like MI and cardiomyopathy through the use of different cell-based therapies.

### Mesenchymal stem cells (MSCs)

The BM, heart, Wharton’s jelly, and adipose tissue are among the prime sources of MSCs [15, 16]. MSCs offer ease of isolation, ex vivo growth, in vitro proliferation, and immune-privileged properties, which is why their use in clinical trials is expanding rapidly [17]. According to the POSEIDON clinical trial on MSC transplantation, allogeneic MSCs are safe and as effective as autologous MSCs [18]. Notably, the TAC-HFT trial revealed the twofold effectiveness of MSCs relative to BM-derived mononuclear cells (BM-MNCs) [19]. Accordingly, MSCs appear to be an excellent candidate for cardiac regeneration trials. Few studies have used MSCs from Wharton’s jelly but the results from both clinical and preclinical studies for this resource are promising [20].

### Cell-based therapy in acute myocardial infarction (AMI)

To date, BM-MNCs have been used in the majority of research on cell-based therapy following AMI. The TIME trials established that the optimal time for cell implantation following AMI is within 3–7 days [21, 22]. Fisher et a., in a meta-analysis, proved that BM-MNCs augment the left ventricular ejection fraction (LVEF) following AMI by roughly 2.72%, yielding benefits both in terms of survival and function in AMI patients younger than 55 years of age with LVEF < 37% [23].

Trials involving the use of MSCs in patients following AMI have shown promising yet controversial results. Gao and coworkers conducted the largest clinical trial in this regard with 116 patients, demonstrating that umbilical cord-derived Wharton’s jelly MSCs (WJ-MSCs) led to an almost five percent improvement in the LVEF [24]. This figure was also confirmed in a meta-analysis for those who receive the cells in the first 10 days after AMI with an improvement of around at 5.74% [25]. These are in agreement with the findings of the TAC-HFT trial, which indicated the roughly twofold effectiveness of MSCs relative to BM-MNCs [19]. Also, a head-to-head comparison of BM-MNCs with MSCs in a meta-analysis showed similar findings (BM-MNC= 3.07%, vs MSCs = 5.65%) [26].

### BAMI trial

For over two decades, autologous cell-based treatments have been assessed in managing cardiovascular diseases through preclinical and clinical studies. However, phase III trials have been infrequent. Furthermore, the phase II trials have involved different methodologies in terms of the type of stem cells and the method and timing of delivery.

The BAMI trial was the first phase III trial conducted to clarify whether or not post-MI intracoronary transplantation of BM-MNCs would reduce all-cause mortality. Although the trial was designed to involve 3000 patients, it was stopped prematurely after the enrollment of 375 patients. Among them, 185 received BM-MNCs (intracoronary infusion) 2–8 days following primary percutaneous coronary intervention (PPCI), and the remaining 190 patients received optimal medical therapy as the control group. All-cause mortality after 2 years was 3.26% [*n*=6; 95% confidence interval (CI): 1.48–7.12%] with BM-MNCs compared to 3.82% (*n*=7; 95% CI: 1.84–7.84%) with optimal medical therapy. The main reason behind such results was that mortality was much lower than expected at the time of study design. At the start of the project in 2011, the literature held that following an AMI, the mortality rate from all causes after 2 years would be approximately 12% among those with an LVEF ≤ 45% post-reperfusion therapy [3]. However, the researchers noticed a 3.85% mortality rate while conducting the study, reflecting the evolution of primary angioplasty procedures in those years. Importantly, the investigators noticed that only five patients (2.7%, 95% CI: 1.0–5.9%) who received BM-MNCs were hospitalized due to HF during the 2 years of follow-up compared with 15 patients (8.1%, CI: 4.7–12.5%) who received optimal medical therapy (HR: 0.33, 95% CI: 0.12–0.88), representing the sole clinical benefit observed. BAMI showed us that taking mortality as an endpoint for stem cell therapy trials may be difficult to achieve as the primary endpoint in trials with medium sample sizes and the best clinical endpoint to assess is HF incidence. In a recent meta-analysis, it was again shown that injection of BM-MNCs was associated with a lower risk of composite end points of hospitalization for congestive heart failure (CHF), re-infarction, and cardiac-related mortality (91/1191 vs. 111/812, RR = 0.643, 95% CI = 0.489 to 0.845, *p* = 0.002). This effect was derived from both reduction of CHF (47/1220 vs. 62/841, RR = 0.568, 95% CI = 0.382 to 0.844, *p* = 0.005) and re-infarction rate (23/1159 vs. 30/775, RR = 0.583, 95% CI = 0.343 to 0.991, *p* = 0.046), but not cardiac-related mortality (28/1290 vs. 31/871, RR = 0.722, 95% CI = 0.436 to 1.197, *p* = 0.207) [27].

### Hypothesis generation

Since the efficacy of MSCs is higher than BM-MNCs after AMI in the improvement of LVEF, it would be probable that these cells may have a better clinical effect as well. However, no study has evaluated the impact of the transplantation of MSCs on a clinical endpoint such as HF.

## Materials and methods

### Study design

**Fig. 1:**
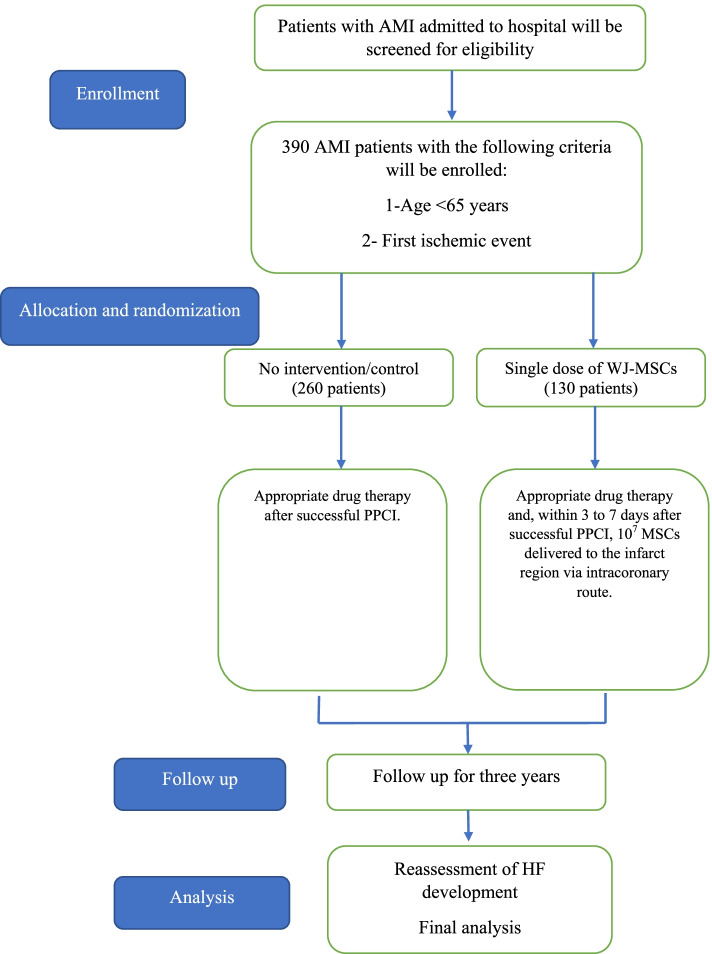
SPIRIT flow diagram of the study

A randomized, multicenter, single-blinded phase III trial will be conducted to assess whether the intracoronary infusion of umbilical cord WJ-MSCs demonstrates a superior effect in reducing HF incidence following AMIs compared to standard treatment. The Ethics Committee of Shiraz University of Medical Sciences approved the study protocol (code: IR.SUMS.REC.1400.409). The trial is registered with https://clinicaltrial.gov under the code NCT05043610. This protocol was conceived following the Standard Protocol Items: Recommendations for Interventional Trials (SPIRIT) guidelines (Online supplement 1). Figure 1 depicts the SPIRIT flow diagram of the study.

### Sample size

Since the primary outcome of this study is to compare the incidence of HF and considering the one-year incidence rate of 1.3% and 4% in the BAMI trial [3], it is predicted that in a 3-year period follow-up of considered for the present study, the incidence rate of this indicator will be 3.9% and 12% in intervention and control groups respectively. According to the formula $\boldsymbol{n}=\frac{\frac{\mathbf{1}+\boldsymbol{R}}{\boldsymbol{R}}\overline{\ \boldsymbol{P}}\Big(\mathbf{1}-\overline{\boldsymbol{P}\Big)}{\left({\boldsymbol{t}}_{\boldsymbol{\alpha}, \boldsymbol{\nu}}+{\boldsymbol{t}}_{\boldsymbol{\beta} \left(\mathbf{1}\right),\boldsymbol{\nu}}\right)}^{\mathbf{2}}}{{\left(\boldsymbol{\partial}\right)}^{\mathbf{2}}}$, and the enrolment ratio of 2:1 in case and control groups, and considering the first type error of 5% and the power of 80%, the required sample size is approximately 220 people in control and 118 people in case group***s***, which makes a total of 328 people. To increase the accuracy of sampling and the possibility of using regression models, the sample size was increased to 390 people. The sample size is approximate and the above formula is summarized. In the above formula $\overline{P}$, the weighted average of the values in the two groups is used, and the *t* values are equal to the 0.8 and 0.975 percentiles of the standard normal distribution and equal to 1.96 and 84. The denominator of the fraction (∂) is the difference of two amounts. R is the ratio of two groups, which is 2 here.

### Study participants

A total of 390 patients with a history of an anterior ST-elevation MI (STEMI) treated successfully with PPCI 3–7 days earlier will be enrolled. Patients must be below 65 years old and must have severely impaired function of the left ventricle (LV), represented by an LVEF of < 40%. The participating hospitals will be the Al-Zahra Heart, Namazee, and Faghihi hospitals of Shiraz, Iran.

The inclusion criteria are as follows:

1. Age (years): 18 to 65
2. Either gender
3. First MI in the preceding 3 to 7 days
4. Post-AMI LVEF < 40%
5. Negative pregnancy test (for women of reproductive age)
6. Written informed consent

The exclusion criteria are as follows:

1. A history of any prior cardiac conditions (valvular, ischemic, or congenital disorders)
2. Regional wall motion abnormalities outside the region of the infarction
3. LV dysfunction due to other etiologies like non-ischemic cardiomyopathy, anthracycline use, or ethanol abuse (> 6 oz./day regularly)
4. Poor echocardiography window
5. Active infection, malignancy, or autoimmune disease

### Randomization and blinding

Randomization will be done via permuted block randomization through a web-based service (https://www.sealedenvelope.com/randomisation/simulation/). A block size of 6 will be considered. Two groups with a 2:1 proportion will be formed, where only one will receive an intracoronary infusion of WJ-MSCs besides the conventional therapy provided to both groups. Those who assess the study outcomes will remain unaware of the allocation (single-blind).

### Intervention

This study will use cGMP-certified clinical-grade human WJ-MSCs (Cell Tech Pharmed Co. Ltd., Tehran, Iran). The cells will be transferred to the hospital on the same day as the infusion and are to be suspended in normal saline (0.9%). Each batch will be analyzed and certificated by a qualified individual, ensuring that the cells comply with the product specifications. Standard operational protocols will be followed during shipment and handling.

In the intervention group, all 130 patients will receive a single intracoronary infusion of 10^7^ WJ-MSCs alongside the conventional treatment that will be provided to the same number of patients in the control group. Patients in the intervention group will be taken to the cardiac catheterization lab, where the infusion of 10^7^ WJ-MSCs will be done through the intracoronary route. A bolus heparin dose (weight-based) is to be administered to patients with an activated clotting time below 200 seconds.

For catheterization, we will insert a therapeutic 6 Fr guiding catheter into the left coronary artery. Following the infusion of nitroglycerin (200 μg) through the catheter, we will assess the left anterior descending (LAD) artery and document the TIMI flow. A 0.014-inch soft-tipped guidewire wire will be inserted into the LAD at the distal edge of the stent. After passing through an over-the-wire balloon to the stented area, the balloon will be inflated until achieving occlusion. Following the removal of the guiding wire, we will connect an infusion syringe to the infusion catheter. WJ-MSCs will be infused at a rate of 2.5 ml/min and the total sample volume would be 7.5 cc. Low-pressure inflation (2–4 bar) will be performed to achieve occlusion with the balloon catheter, with complete coronary artery occlusion being ensured ahead of cell infusion through the use of dye. After the infusion of each third of the cells, we will pause the infusion, and TIMI coronary flow will be assessed with the contrast agent before the resumption of cell infusion. Once the cells are delivered across the three portions, we will place the coronary flow wire via the microinfusion catheter. No methods were used to increase adherence for this intervention as it was a one-off treatment with no need for continued adherence.

The patients in both control and intervention groups will also receive standard Guideline directed medical therapy for acute myocardial infarction, consisting of Aspirin, ticagrelor, rosuvastatin, valsartan, and bisoprolol. Also based on the situation eplerenone and ICD insertion would be considered.

The original design of the study included a sham procedure for the control group but the local ethical committee refused that due to its invasive nature and obliged us to omit the sham procedure from the study protocol.

### Follow-up and endpoints

Patients will receive daily visits from a cardiologist during hospitalization. The results of all physical examinations will be recorded, and patients will be monitored for early manifestations signaling arrhythmia, pulmonary embolism, or coronary artery injury. Blood tests will be done to measure fasting blood sugar, complete blood count, C-reactive protein, urea and electrolytes, liver function test, creatine kinase, and cardiac troponin T. An electrocardiogram (ECG) will also be obtained. Prior to the MSC infusion process, the cardiac evaluation will be completed using echocardiography. The initial EF will be established according to the wall motion score and Simpson’s rule. After the MSCs are delivered, once stable, a beta-blocker, angiotensin-converting enzyme (ACE) inhibitor, aldosterone antagonist, aspirin, ticagrelor, statin, and glyceryl trinitrate (spray or tablets) will be prescribed for the patient to use at home. A cardiac rehabilitation program will also be completed. Subsequent visits will be at ten days after discharge and then every three months when an ECG and blood tests will be requested. Echocardiography will be done during the six-month follow-up and the final visit, facilitating the evaluation of LV systolic function.

Our primary endpoint to assess the efficacy of the intervention will be the incidence of HF. Secondary endpoints include the improvement in LV function (through calculation of LVEF) after six months and after three years alongside echocardiographic changes in the left ventricular mass, left ventricular end-diastolic volume, left ventricular end-systolic diameter, and global longitudinal strain (measured via automated formulas in standard views) indices.

Data will be entered, encoded, became secure, and stored in a local database.

### Adjudication of study measures

Before statistical analysis, adjudication of all measurements will be done by an experienced cardiology department member excluded from the research group. The adjudicator will assess the quality of each measurement and will exclude those with inadequate quality from the analysis, where they will be regarded as missing. An independent, blinded safety committee will evaluate potential major adverse cardiac events (MACEs). Once the adjudication process is complete, the finalized database will be unblinded.

### Statistical analysis

Data will be kept anonymous until analysis, which is to be performed by an independent statistician external to the research group. Continuous variables will be summarized using the mean and standard deviation, while frequencies and percentages will be given for categorical data. The analysis will follow the intention-to-treat approach. Two-sided P-values will be used and a *P*<0.05 will be considered as significant.

The primary endpoint of the study which is the incidence of heart failure will be compared between groups using Cox regression analysis.

We will consider the EF to have improved significantly if a minimum increment of 3% is achieved after six months. The EF, as the secondary outcome, will be compared between the study groups using the independent t-test.

The baseline characteristics of the two study groups will also be compared using independent sample *T*-test.

Safety will be compared between the two groups according to the occurrence of MACEs (death, recurrent AMI, ICD insertion, non-target vessel revascularization, etc.) and serious adverse events (SAEs) using COX regression analysis. These events will be followed over time with Kaplan-Meier curves, which will allow us to understand their patterns.

### Adverse events

Adverse events will be reported by the study’s executive committee to an independent Data and Safety and Monitoring Board (DSMB). The DSMB will have the authority to stop the trial early if patient safety is compromised or if the primary research objective is met. If the presumed 5% statistically significant difference in EF was achieved, it would be considered as reaching the primary objective of the study. A stringent statistical threshold would be used as the stopping rule. All safety issues (unanticipated SAEs, mortality, intracoronary infusion complications, severe arrhythmias, etc.) will be monitored by the DSMB, and the DSMB statistician will report the occurrence of safety issues in each study group quarterly. All deaths will be reported. Auditing would be done by DSMB on a 6-month interval and is independent of investigators.

### Ethical considerations

We discussed all ethical issues with the Institutional Review Board of Shiraz University of Medical Sciences, which ultimately approved the study protocol (IR.SUMS.REC.1400.409). Informed consent will be obtained once patients are clinically stable and sedatives or strong analgesics do not alter their consciousness. Importantly, the use of low balloon inflation pressure and divided (three-part) infusions will prevent complications related to intracoronary cell infusion. The principles of the *Declaration of Helsinki* will be upheld throughout this study.

## Discussion

Currently, the primary focus of post-AMI treatment is to prevent remodeling and avert any further loss of myocytes [5]. However, a revolution can potentially be achieved by regenerative medicine, aiming to restore cardiac function by inhibiting and even reversing the process of remodeling through the use of stem cells [24]. Even though some investigations were not very promising in this regard [8], other studies have shown that stem cell therapy may be of value in certain populations.

Although a Cochrane meta-analysis revealed that the LVEF of young AMI patients does not increase following BM-MNC therapy, survival and functional benefits may be present [23]. Importantly, research with MSCs has yielded more promising results, with the TAC-HFT trial indicating the roughly twofold higher efficacy of MSCs relative to BM-MNCs [19]. According to meta-analyses of the various clinical trials, MSCs can improve the LVEF by 5.72% [25], while BM-MNCs can achieve an inferior improvement of 3.07% [26].

Currently, scientists are yet to understand the exact mechanisms behind the therapeutic impact of stem cells, especially MSCs. However, the most commonly suggested mechanism is paracrine signaling, where the implanted stem cells alter the activity of the nearby cells in the heart via mediators like cytokines [28]. Also, it is still unclear whether or not the mechanical improvements in LV function after MSC transplantation would be translated into a clinical benefit by reducing major cardiovascular events. Our trial provides essential insights into the field by including selected patients who develop reduced LVEFs after AMIs.

Our trial, enrolling 390 patients, would be the largest clinical trial ever conducted in the field of regenerative medicine on myocardial infarction. We hope it would help clarify whether MSC transplantation is clinically useful or not.

### Trial status

We began enrolling patients in Jan 2021 and expect to finish the recruitment process by Nov 2022. Data will be collected until Nov 2024. Thereafter, the analysis will be conducted. Results are expected to be ready by Dec 2024. We will prepare and submit the related manuscript following the CONSORT guidelines. This study is registered with *ClinicalTrials.gov* under the code NCT05043610. This protocol (version 1) was approved in Oct 2020. Changes in protocol will be submitted to the registration site.

## Supplementary Information

**Additional file 1.** SPIRIT 2013 Checklist: Recommended items to address in a clinical trial protocol and related documents.

## Data Availability

Data will be made available upon reasonable request following the completion of the study.

## Abbreviations

AMI: Acute myocardial infarction
BM: Bone marrow
BM-MNCs: Bone marrow-derived mononuclear cells
DSMB: Data and Safety and Monitoring Board
EF: Ejection fraction
HF: Heart failure
LVEF: Left ventricular ejection fraction
MACEs: Major adverse cardiac events
MCSs: Mesenchymal stem cells
MI: Myocardial infarction
SAEs: Serious adverse events
WJ-MSCs: Wharton’s jelly mesenchymal stem cells
